# 3-[4-(Dimethylamino)phenyl]-1,5-di­phenylpentane-1,5-dione

**DOI:** 10.1107/S1600536808023866

**Published:** 2008-07-31

**Authors:** Qing-Peng He, Xiao-Qiang Qin, Xiao Wang, Qiu-Lan Shi, Yong Wang

**Affiliations:** a Department of Chemistry, Liaocheng University, Liaocheng 252059, People’s Republic of China; b Department of Chemistry, Weifang Medical University, Weifang Shandong Province, 261053, People’s Republic of China; c No.1 Middle School of Liaocheng, Liaocheng 252059, People’s Republic of China

## Abstract

The asymmetric unit of the title compound, C_25_H_25_NO_2_, contains two independent mol­ecules. The crystal packing exhibits weak inter­molecular C—H⋯O, C—H⋯π and π–π inter­actions.

## Related literature

For crystal structures of related compounds, see Das *et al.* (1994 ▶); Huang *et al.* (2006 ▶). For general background, see Bose *et al.* (2004 ▶). 
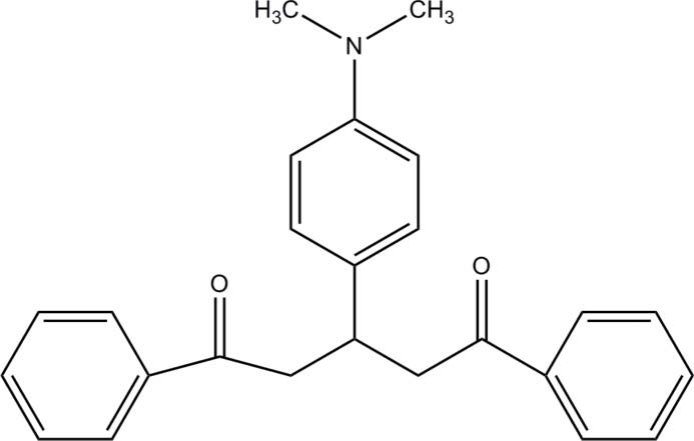


## Experimental

### 

#### Crystal data

C_25_H_25_NO_2_
*M* *_r_* = 371.46
Triclinic, 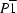

*a* = 9.926 (1) Å
*b* = 11.3749 (14) Å
*c* = 18.853 (2) Å
α = 90.443 (10)°
β = 94.782 (10)°
γ = 99.862 (2)°
*V* = 2089.3 (4) Å^3^
*Z* = 4
Mo *K*α radiation
μ = 0.07 mm^−1^
*T* = 298 (2) K
0.49 × 0.40 × 0.29 mm

#### Data collection

Bruker SMART CCD area-detector diffractometer
Absorption correction: multi-scan (*SADABS*; Sheldrick, 1996 ▶) *T*_min_ = 0.965, *T*_max_ = 0.979
11011 measured reflections
7259 independent reflections
3113 reflections with *I* > 2σ(*I*)
*R*_int_ = 0.032

#### Refinement

*R*[*F* ^2^ > 2σ(*F* ^2^)] = 0.052
*wR*(*F* ^2^) = 0.139
*S* = 0.99
7259 reflections
505 parameters
H-atom parameters constrained
Δρ_max_ = 0.17 e Å^−3^
Δρ_min_ = −0.15 e Å^−3^

### 

Data collection: *SMART* (Siemens, 1996 ▶); cell refinement: *SAINT* (Siemens, 1996 ▶); data reduction: *SAINT*; program(s) used to solve structure: *SHELXS97* (Sheldrick, 2008 ▶); program(s) used to refine structure: *SHELXS97* (Sheldrick, 2008 ▶); molecular graphics: *SHELXTL* (Sheldrick, 2008 ▶); software used to prepare material for publication: *SHELXTL*.

## Supplementary Material

Crystal structure: contains datablocks I, global. DOI: 10.1107/S1600536808023866/cv2435sup1.cif

Structure factors: contains datablocks I. DOI: 10.1107/S1600536808023866/cv2435Isup2.hkl

Additional supplementary materials:  crystallographic information; 3D view; checkCIF report

## Figures and Tables

**Table 1:** Centroid⋯centroid distance (Å)

*Cg*1⋯*Cg*1^i^	3.773 (4)

Symmetry code: (i) 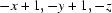. *Cg*1 is the centroid of atoms C45–C50.

**Table 2:** Hydrogen-bond geometry (Å, °)

*D*—H⋯*A*	*D*—H	H⋯*A*	*D*⋯*A*	*D*—H⋯*A*
C46—H46⋯O1	0.93	2.51	3.436 (4)	172
C23—H23⋯*Cg*2^ii^	0.93	2.67	3.535 (4)	155

Symmetry code: (ii) 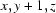. *Cg*2 is the centroid of atoms C2–C7.

**Fig. 1.**
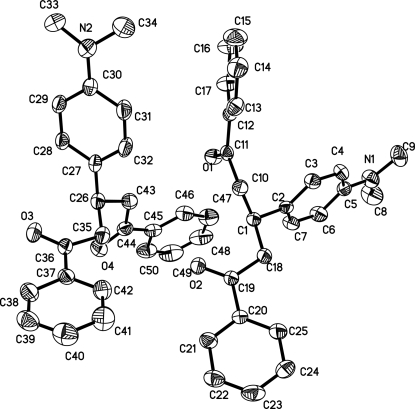
Two independent molecules of (I) with the atom numbering scheme and 30% probability displacement ellipsoids. Hydrogen atoms are omitted for clarity

## supplementary crystallographic information

### Comment

The "Grindstone Chemistry" method for conducting exothermic reactions in the solvent-free mode has been recently published (Bose *et al.*, 2004). When this protocol was applied to the Pechmann synthesis on a multi-molar scale, the expected coumarins were obtained in a few minutes as pure products in high yield by solvent-free grinding. Since this reaction proved to be exothermic, we tested energy-saving procedures developed in our laboratory for the preparation of 1,5-diketones starting from the fragrant aldehydes and fragrant ketones in the presence of NaOH under solvent-free conditions. Using this method, we obtained the title compound, (I). Herewith we present its crystal structure.

In (I), all bond lengths and angles are normal and correspond to those observed in 1,3,5-triphenyl-pentane-1,5-diketone (Das *et al.*, 1994) and 1,5-diphenyl-3-(2-pyridyl)pentane-1,5-dione (Huang *et al.*cv2222435, 2006). The asymmetric unit of (I) contains two independent molecules (Fig. 1) with different conformations - the dihedral angles formed by two phenyl rings in each molecule are 85.48 (7) and 71.26 (7)°, respectively.

The weak intermolecular π–π (Table 1) and C—H···π (Table 2) interactions, and C—H···O hydrogen bonds (Table 2) contribute to the crystal packing stabilization.

### Experimental

Acetophenone (6.25 mmol) and 4-(dimethylamino)benzaldehyde (3.125 mmol), NaOH (6.25 mmol) were aggregated with glass paddle in an open flask. The resulting mixture was washed with water for several times for removing NaOH, and recrystalized from ethanol, and afforded the title compound as a crystalline solid. Elemental analysis: calculated for C_25_ H_25_ NO_2_: C 80.83, H 6.78, N 3.77%; Found: C80.88, H 6.83, N3.65%.

### Refinement

All H atoms were positioned geometrically (C—H 0.93–0.98 Å) and refined using a riding model, with *U*_iso_(H) = 1.2-1.5*U*_eq_(C).

### Crystal data

**Table:**

C_25_H_25_NO_2_	*Z* = 4
*M**_r_* = 371.46	*F*_000_ = 792
Triclinic, *P*1	*D*_x_ = 1.181 Mg m^−^^3^
*a* = 9.9260 (10) Å	Mo *K*α radiation λ = 0.71073 Å
*b* = 11.3749 (14) Å	Cell parameters from 1876 reflections
*c* = 18.853 (2) Å	θ = 2.3–21.9º
α = 90.4430 (10)º	µ = 0.07 mm^−^^1^
β = 94.7820 (10)º	*T* = 298 (2) K
γ = 99.862 (2)º	Needle, orange
*V* = 2089.3 (4) Å^3^	0.49 × 0.40 × 0.29 mm

### Data collection

**Table:**

Bruker SMART CCD area-detector diffractometer	7259 independent reflections
Radiation source: fine-focus sealed tube	3113 reflections with *I* > 2σ(*I*)
Monochromator: graphite	*R*_int_ = 0.032
*T* = 298(2) K	θ_max_ = 25.0º
phi and ω scans	θ_min_ = 1.8º
Absorption correction: multi-scan(SADABS; Sheldrick, 1996)	*h* = −11→11
*T*_min_ = 0.965, *T*_max_ = 0.979	*k* = −13→11
11011 measured reflections	*l* = −21→22

### Refinement

**Table:**

Refinement on *F*^2^	Secondary atom site location: difference Fourier map
Least-squares matrix: full	Hydrogen site location: inferred from neighbouring sites
*R*[*F*^2^ > 2σ(*F*^2^)] = 0.052	H-atom parameters constrained
*wR*(*F*^2^) = 0.139	*w* = 1/[σ^2^(*F*_o_^2^) + (0.0484*P*)^2^] where *P* = (*F*_o_^2^ + 2*F*_c_^2^)/3
*S* = 0.99	(Δ/σ)_max_ = 0.001
7259 reflections	Δρ_max_ = 0.17 e Å^−^^3^
505 parameters	Δρ_min_ = −0.15 e Å^−^^3^
Primary atom site location: structure-invariant direct methods	Extinction correction: none

### Special details

**Table:**

Geometry. All e.s.d.'s (except the e.s.d. in the dihedral angle between two l.s. planes) are estimated using the full covariance matrix. The cell e.s.d.'s are taken into account individually in the estimation of e.s.d.'s in distances, angles and torsion angles; correlations between e.s.d.'s in cell parameters are only used when they are defined by crystal symmetry. An approximate (isotropic) treatment of cell e.s.d.'s is used for estimating e.s.d.'s involving l.s. planes.
Refinement. Refinement of *F*^2^ against ALL reflections. The weighted *R*-factor *wR* and goodness of fit *S* are based on *F*^2^, conventional *R*-factors *R* are based on *F*, with *F* set to zero for negative *F*^2^. The threshold expression of *F*^2^ > σ(*F*^2^) is used only for calculating *R*-factors(gt) *etc*. and is not relevant to the choice of reflections for refinement. *R*-factors based on *F*^2^ are statistically about twice as large as those based on *F*, and *R*- factors based on ALL data will be even larger.

### Fractional atomic coordinates and isotropic or equivalent isotropic displacement parameters (Å^2^)

**Table:**

	*x*	*y*	*z*	*U*_iso_*/*U*_eq_	
N1	−0.2518 (3)	0.1016 (2)	0.07384 (14)	0.0746 (8)	
N2	0.7107 (3)	0.3289 (3)	0.54492 (15)	0.0770 (8)	
O1	0.3269 (2)	0.29812 (16)	0.24964 (10)	0.0591 (6)	
O2	0.3520 (2)	0.69008 (17)	0.22151 (11)	0.0672 (6)	
O3	0.9223 (2)	0.7410 (2)	0.31919 (13)	0.0890 (8)	
O4	0.7803 (2)	0.5520 (2)	0.12546 (11)	0.0851 (8)	
C1	0.1699 (3)	0.4704 (2)	0.20068 (15)	0.0484 (8)	
H1	0.2518	0.4698	0.1751	0.058*	
C2	0.0584 (3)	0.3716 (2)	0.16849 (15)	0.0442 (7)	
C3	−0.0406 (3)	0.3083 (2)	0.20687 (15)	0.0538 (8)	
H3	−0.0384	0.3247	0.2554	0.065*	
C4	−0.1433 (3)	0.2212 (2)	0.17590 (15)	0.0548 (8)	
H4	−0.2084	0.1811	0.2039	0.066*	
C5	−0.1514 (3)	0.1923 (2)	0.10427 (15)	0.0504 (8)	
C6	−0.0526 (3)	0.2565 (3)	0.06505 (15)	0.0638 (9)	
H6	−0.0549	0.2408	0.0164	0.077*	
C7	0.0484 (3)	0.3429 (3)	0.09681 (15)	0.0586 (9)	
H7	0.1129	0.3839	0.0688	0.070*	
C8	−0.2579 (4)	0.0717 (3)	0.00048 (18)	0.1051 (14)	
H8A	−0.2723	0.1397	−0.0271	0.158*	
H8B	−0.3322	0.0068	−0.0112	0.158*	
H8C	−0.1731	0.0483	−0.0100	0.158*	
C9	−0.3612 (3)	0.0485 (3)	0.11383 (19)	0.0898 (12)	
H9A	−0.3261	0.0017	0.1509	0.135*	
H9B	−0.4293	−0.0020	0.0830	0.135*	
H9C	−0.4018	0.1099	0.1345	0.135*	
C10	0.2091 (3)	0.4528 (2)	0.27920 (14)	0.0517 (8)	
H10A	0.2778	0.5200	0.2969	0.062*	
H10B	0.1288	0.4524	0.3053	0.062*	
C11	0.2644 (3)	0.3393 (2)	0.29382 (15)	0.0438 (7)	
C12	0.2454 (3)	0.2790 (3)	0.36266 (14)	0.0455 (7)	
C13	0.2109 (3)	0.3365 (3)	0.42188 (16)	0.0600 (9)	
H13	0.1970	0.4152	0.4188	0.072*	
C14	0.1968 (3)	0.2784 (4)	0.48502 (18)	0.0782 (11)	
H14	0.1761	0.3180	0.5250	0.094*	
C15	0.2138 (4)	0.1613 (4)	0.48865 (19)	0.0938 (13)	
H15	0.2028	0.1211	0.5311	0.113*	
C16	0.2465 (4)	0.1033 (3)	0.4305 (2)	0.0950 (13)	
H16	0.2574	0.0238	0.4334	0.114*	
C17	0.2635 (3)	0.1622 (3)	0.36776 (17)	0.0728 (10)	
H17	0.2873	0.1229	0.3285	0.087*	
C18	0.1245 (3)	0.5911 (2)	0.18845 (16)	0.0560 (8)	
H18A	0.0851	0.5922	0.1397	0.067*	
H18B	0.0524	0.5976	0.2192	0.067*	
C19	0.2346 (3)	0.6992 (2)	0.20144 (15)	0.0475 (8)	
C20	0.1967 (3)	0.8179 (2)	0.18730 (14)	0.0469 (7)	
C21	0.2981 (3)	0.9170 (3)	0.19303 (14)	0.0594 (9)	
H21	0.3884	0.9077	0.2049	0.071*	
C22	0.2693 (4)	1.0295 (3)	0.18159 (17)	0.0695 (10)	
H22	0.3398	1.0952	0.1851	0.083*	
C23	0.1376 (5)	1.0446 (3)	0.16508 (18)	0.0801 (11)	
H23	0.1175	1.1209	0.1584	0.096*	
C24	0.0341 (4)	0.9469 (3)	0.15827 (19)	0.0836 (11)	
H24	−0.0560	0.9570	0.1464	0.100*	
C25	0.0640 (3)	0.8339 (3)	0.16907 (17)	0.0682 (9)	
H25	−0.0062	0.7680	0.1640	0.082*	
C26	0.7350 (3)	0.5403 (3)	0.26971 (15)	0.0563 (8)	
H26	0.8298	0.5466	0.2572	0.068*	
C27	0.7282 (3)	0.4872 (2)	0.34239 (16)	0.0505 (8)	
C28	0.8433 (3)	0.4613 (2)	0.38126 (17)	0.0553 (8)	
H28	0.9278	0.4806	0.3624	0.066*	
C29	0.8387 (3)	0.4083 (3)	0.44654 (17)	0.0580 (9)	
H29	0.9193	0.3921	0.4701	0.070*	
C30	0.7166 (3)	0.3785 (3)	0.47788 (17)	0.0551 (8)	
C31	0.6001 (3)	0.4050 (3)	0.43978 (17)	0.0716 (10)	
H31	0.5155	0.3865	0.4586	0.086*	
C32	0.6080 (3)	0.4582 (3)	0.37451 (17)	0.0678 (10)	
H32	0.5278	0.4753	0.3509	0.081*	
C33	0.8266 (4)	0.2791 (3)	0.57485 (19)	0.0954 (12)	
H33A	0.8411	0.2150	0.5446	0.143*	
H33B	0.8089	0.2494	0.6213	0.143*	
H33C	0.9071	0.3399	0.5786	0.143*	
C34	0.5808 (4)	0.2732 (4)	0.5674 (2)	0.1185 (16)	
H34A	0.5191	0.3297	0.5657	0.178*	
H34B	0.5934	0.2463	0.6153	0.178*	
H34C	0.5430	0.2062	0.5364	0.178*	
C35	0.6987 (3)	0.6660 (3)	0.26728 (16)	0.0640 (9)	
H35A	0.6814	0.6865	0.2179	0.077*	
H35B	0.6145	0.6651	0.2900	0.077*	
C36	0.8079 (3)	0.7616 (3)	0.30282 (15)	0.0579 (9)	
C37	0.7755 (4)	0.8824 (3)	0.31653 (14)	0.0547 (8)	
C38	0.8810 (4)	0.9747 (3)	0.33986 (16)	0.0742 (10)	
H38	0.9710	0.9610	0.3442	0.089*	
C39	0.8547 (5)	1.0854 (3)	0.35654 (18)	0.0856 (12)	
H39	0.9265	1.1458	0.3724	0.103*	
C40	0.7232 (5)	1.1073 (3)	0.34998 (19)	0.0929 (13)	
H40	0.7055	1.1824	0.3619	0.111*	
C41	0.6170 (4)	1.0184 (4)	0.32569 (19)	0.0912 (12)	
H41	0.5274	1.0332	0.3206	0.109*	
C42	0.6445 (4)	0.9068 (3)	0.30883 (16)	0.0691 (10)	
H42	0.5726	0.8470	0.2919	0.083*	
C43	0.6433 (3)	0.4592 (3)	0.21342 (14)	0.0594 (9)	
H43A	0.6464	0.3768	0.2251	0.071*	
H43B	0.5494	0.4715	0.2156	0.071*	
C44	0.6818 (3)	0.4790 (3)	0.13862 (16)	0.0596 (9)	
C45	0.5958 (4)	0.4051 (3)	0.07981 (15)	0.0560 (8)	
C46	0.4716 (4)	0.3347 (3)	0.09015 (16)	0.0652 (9)	
H46	0.4407	0.3293	0.1354	0.078*	
C47	0.3927 (4)	0.2721 (3)	0.0337 (2)	0.0849 (12)	
H47	0.3092	0.2247	0.0411	0.102*	
C48	0.4374 (5)	0.2799 (3)	−0.0329 (2)	0.0950 (14)	
H48	0.3836	0.2386	−0.0709	0.114*	
C49	0.5601 (5)	0.3476 (4)	−0.0439 (2)	0.0997 (14)	
H49	0.5902	0.3514	−0.0893	0.120*	
C50	0.6408 (4)	0.4110 (3)	0.01199 (19)	0.0811 (11)	
H50	0.7247	0.4572	0.0041	0.097*	

### Atomic displacement parameters (Å^2^)

**Table:**

	*U*^11^	*U*^22^	*U*^33^	*U*^12^	*U*^13^	*U*^23^
N1	0.076 (2)	0.073 (2)	0.0634 (19)	−0.0123 (17)	−0.0067 (16)	−0.0099 (15)
N2	0.069 (2)	0.091 (2)	0.068 (2)	0.0095 (18)	−0.0050 (17)	0.0116 (17)
O1	0.0684 (15)	0.0540 (13)	0.0604 (13)	0.0197 (11)	0.0191 (11)	0.0048 (10)
O2	0.0488 (14)	0.0509 (13)	0.0990 (17)	0.0065 (11)	−0.0074 (13)	0.0100 (11)
O3	0.0569 (16)	0.0780 (17)	0.124 (2)	0.0050 (13)	−0.0276 (15)	0.0143 (14)
O4	0.0693 (17)	0.0997 (19)	0.0812 (16)	−0.0029 (14)	0.0096 (13)	0.0295 (14)
C1	0.0450 (19)	0.0388 (18)	0.061 (2)	0.0068 (15)	0.0029 (15)	0.0055 (14)
C2	0.0478 (19)	0.0331 (17)	0.0509 (19)	0.0072 (14)	−0.0012 (15)	0.0051 (14)
C3	0.061 (2)	0.0469 (19)	0.0516 (18)	0.0044 (17)	0.0039 (17)	−0.0019 (15)
C4	0.052 (2)	0.053 (2)	0.056 (2)	−0.0037 (16)	0.0100 (16)	0.0003 (16)
C5	0.051 (2)	0.0440 (19)	0.054 (2)	0.0041 (16)	−0.0040 (16)	−0.0008 (16)
C6	0.079 (3)	0.061 (2)	0.0468 (19)	0.001 (2)	0.0007 (18)	−0.0006 (17)
C7	0.069 (2)	0.051 (2)	0.053 (2)	−0.0005 (18)	0.0084 (17)	0.0082 (16)
C8	0.129 (4)	0.096 (3)	0.072 (3)	−0.021 (3)	−0.012 (2)	−0.021 (2)
C9	0.070 (3)	0.075 (3)	0.113 (3)	−0.015 (2)	0.000 (2)	−0.015 (2)
C10	0.053 (2)	0.0395 (18)	0.062 (2)	0.0083 (15)	0.0004 (16)	−0.0042 (15)
C11	0.0385 (18)	0.0359 (18)	0.0550 (19)	0.0024 (14)	0.0009 (15)	−0.0048 (15)
C12	0.0433 (19)	0.0454 (19)	0.0472 (19)	0.0081 (15)	−0.0004 (15)	0.0015 (15)
C13	0.060 (2)	0.068 (2)	0.054 (2)	0.0176 (18)	0.0000 (17)	−0.0043 (18)
C14	0.082 (3)	0.105 (3)	0.050 (2)	0.020 (2)	0.0064 (19)	−0.004 (2)
C15	0.118 (4)	0.106 (4)	0.053 (2)	0.007 (3)	0.007 (2)	0.021 (2)
C16	0.143 (4)	0.067 (3)	0.074 (3)	0.018 (2)	0.002 (3)	0.021 (2)
C17	0.105 (3)	0.055 (2)	0.062 (2)	0.023 (2)	0.007 (2)	0.0072 (18)
C18	0.046 (2)	0.0402 (18)	0.079 (2)	0.0042 (15)	−0.0013 (17)	0.0052 (15)
C19	0.045 (2)	0.0433 (19)	0.0547 (19)	0.0074 (16)	0.0055 (16)	0.0053 (14)
C20	0.050 (2)	0.0403 (19)	0.0510 (18)	0.0101 (16)	0.0035 (16)	0.0072 (14)
C21	0.065 (2)	0.045 (2)	0.065 (2)	0.0048 (18)	−0.0004 (17)	0.0017 (16)
C22	0.087 (3)	0.043 (2)	0.076 (2)	0.007 (2)	0.005 (2)	0.0001 (17)
C23	0.106 (3)	0.047 (2)	0.095 (3)	0.028 (2)	0.020 (3)	0.0124 (19)
C24	0.068 (3)	0.068 (3)	0.124 (3)	0.033 (2)	0.015 (2)	0.022 (2)
C25	0.063 (2)	0.047 (2)	0.096 (3)	0.0117 (18)	0.009 (2)	0.0137 (18)
C26	0.044 (2)	0.060 (2)	0.064 (2)	0.0085 (16)	0.0022 (16)	0.0021 (17)
C27	0.0353 (19)	0.056 (2)	0.059 (2)	0.0082 (15)	0.0001 (16)	−0.0059 (16)
C28	0.038 (2)	0.058 (2)	0.070 (2)	0.0100 (16)	0.0025 (17)	−0.0009 (17)
C29	0.043 (2)	0.060 (2)	0.070 (2)	0.0120 (17)	−0.0063 (18)	−0.0004 (18)
C30	0.049 (2)	0.059 (2)	0.055 (2)	0.0056 (17)	−0.0039 (18)	−0.0038 (16)
C31	0.044 (2)	0.106 (3)	0.062 (2)	0.0048 (19)	0.0057 (18)	0.005 (2)
C32	0.040 (2)	0.100 (3)	0.063 (2)	0.0158 (19)	−0.0046 (18)	0.004 (2)
C33	0.089 (3)	0.105 (3)	0.089 (3)	0.016 (2)	−0.009 (2)	0.035 (2)
C34	0.090 (3)	0.163 (4)	0.101 (3)	0.006 (3)	0.022 (3)	0.051 (3)
C35	0.053 (2)	0.064 (2)	0.071 (2)	0.0066 (18)	−0.0096 (17)	0.0012 (17)
C36	0.049 (2)	0.066 (2)	0.0541 (19)	0.0009 (19)	−0.0065 (17)	0.0113 (17)
C37	0.061 (2)	0.054 (2)	0.0440 (18)	−0.0014 (19)	−0.0017 (17)	0.0060 (15)
C38	0.074 (3)	0.072 (3)	0.067 (2)	−0.010 (2)	−0.0039 (19)	0.0096 (19)
C39	0.106 (4)	0.064 (3)	0.076 (3)	−0.011 (3)	−0.005 (3)	−0.001 (2)
C40	0.122 (4)	0.070 (3)	0.083 (3)	0.008 (3)	0.005 (3)	−0.013 (2)
C41	0.091 (3)	0.080 (3)	0.104 (3)	0.022 (3)	0.007 (2)	−0.018 (2)
C42	0.067 (3)	0.068 (3)	0.069 (2)	0.006 (2)	0.000 (2)	−0.0034 (18)
C43	0.059 (2)	0.064 (2)	0.055 (2)	0.0085 (17)	0.0050 (17)	0.0033 (16)
C44	0.058 (2)	0.058 (2)	0.066 (2)	0.0190 (18)	0.0085 (19)	0.0140 (18)
C45	0.074 (2)	0.053 (2)	0.049 (2)	0.0256 (18)	0.0162 (18)	0.0104 (16)
C46	0.086 (3)	0.055 (2)	0.054 (2)	0.0110 (19)	0.0068 (19)	0.0005 (17)
C47	0.114 (3)	0.063 (2)	0.072 (3)	0.008 (2)	−0.007 (2)	−0.007 (2)
C48	0.162 (5)	0.066 (3)	0.058 (3)	0.031 (3)	−0.009 (3)	−0.005 (2)
C49	0.162 (5)	0.096 (3)	0.052 (3)	0.047 (3)	0.020 (3)	0.001 (2)
C50	0.103 (3)	0.082 (3)	0.068 (3)	0.033 (2)	0.028 (2)	0.019 (2)

### Geometric parameters (Å, °)

**Table:**

N1—C5	1.389 (3)	C23—C24	1.376 (4)
N1—C8	1.416 (4)	C23—H23	0.9300
N1—C9	1.425 (4)	C24—C25	1.381 (4)
N2—C30	1.390 (4)	C24—H24	0.9300
N2—C34	1.435 (4)	C25—H25	0.9300
N2—C33	1.443 (4)	C26—C27	1.503 (4)
O1—C11	1.216 (3)	C26—C43	1.530 (3)
O2—C19	1.217 (3)	C26—C35	1.534 (4)
O3—C36	1.214 (3)	C26—H26	0.9800
O4—C44	1.213 (3)	C27—C32	1.374 (4)
C1—C2	1.519 (3)	C27—C28	1.382 (4)
C1—C10	1.523 (3)	C28—C29	1.376 (4)
C1—C18	1.529 (4)	C28—H28	0.9300
C1—H1	0.9800	C29—C30	1.383 (4)
C2—C3	1.376 (3)	C29—H29	0.9300
C2—C7	1.379 (3)	C30—C31	1.389 (4)
C3—C4	1.381 (3)	C31—C32	1.378 (4)
C3—H3	0.9301	C31—H31	0.9300
C4—C5	1.380 (4)	C32—H32	0.9300
C4—H4	0.9300	C33—H33A	0.9600
C5—C6	1.388 (4)	C33—H33B	0.9600
C6—C7	1.372 (3)	C33—H33C	0.9600
C6—H6	0.9301	C34—H34A	0.9600
C7—H7	0.9300	C34—H34B	0.9600
C8—H8A	0.9600	C34—H34C	0.9600
C8—H8B	0.9600	C35—C36	1.506 (4)
C8—H8C	0.9600	C35—H35A	0.9700
C9—H9A	0.9600	C35—H35B	0.9700
C9—H9B	0.9600	C36—C37	1.489 (4)
C9—H9C	0.9600	C37—C42	1.371 (4)
C10—C11	1.506 (4)	C37—C38	1.390 (4)
C10—H10A	0.9700	C38—C39	1.368 (4)
C10—H10B	0.9700	C38—H38	0.9300
C11—O1	1.216 (3)	C39—C40	1.366 (5)
C11—C12	1.484 (4)	C39—H39	0.9300
C12—C17	1.374 (4)	C40—C41	1.375 (4)
C12—C13	1.386 (4)	C40—H40	0.9300
C13—C14	1.371 (4)	C41—C42	1.384 (4)
C13—H13	0.9300	C41—H41	0.9300
C14—C15	1.372 (5)	C42—H42	0.9300
C14—H14	0.9300	C43—C44	1.500 (4)
C15—C16	1.366 (5)	C43—H43A	0.9700
C15—H15	0.9300	C43—H43B	0.9700
C16—C17	1.372 (4)	C44—C45	1.499 (4)
C16—H16	0.9300	C45—C46	1.380 (4)
C17—H17	0.9300	C45—C50	1.387 (4)
C18—C19	1.503 (3)	C46—C47	1.382 (4)
C18—H18A	0.9700	C46—H46	0.9299
C18—H18B	0.9700	C47—C48	1.364 (5)
C19—C20	1.483 (4)	C47—H47	0.9300
C20—C21	1.374 (3)	C48—C49	1.357 (5)
C20—C25	1.376 (4)	C48—H48	0.9299
C21—C22	1.373 (4)	C49—C50	1.386 (5)
C21—H21	0.9300	C49—H49	0.9298
C22—C23	1.359 (4)	C50—H50	0.9300
C22—H22	0.9300		
			
Cg1···Cg1^i^	3.773 (4)		
			
C5—N1—C8	120.7 (3)	C20—C25—C24	120.6 (3)
C5—N1—C9	120.1 (3)	C20—C25—H25	119.7
C8—N1—C9	118.7 (3)	C24—C25—H25	119.7
C30—N2—C34	119.6 (3)	C27—C26—C43	111.4 (2)
C30—N2—C33	118.6 (3)	C27—C26—C35	112.9 (2)
C34—N2—C33	114.6 (3)	C43—C26—C35	109.9 (2)
C2—C1—C10	112.8 (2)	C27—C26—H26	107.5
C2—C1—C18	109.4 (2)	C43—C26—H26	107.5
C10—C1—C18	111.5 (2)	C35—C26—H26	107.5
C2—C1—H1	107.6	C32—C27—C28	114.9 (3)
C10—C1—H1	107.6	C32—C27—C26	123.1 (3)
C18—C1—H1	107.6	C28—C27—C26	122.0 (3)
C3—C2—C7	115.7 (3)	C29—C28—C27	123.0 (3)
C3—C2—C1	123.5 (3)	C29—C28—H28	118.5
C7—C2—C1	120.8 (3)	C27—C28—H28	118.5
C2—C3—C4	122.3 (3)	C28—C29—C30	121.4 (3)
C2—C3—H3	118.8	C28—C29—H29	119.3
C4—C3—H3	118.8	C30—C29—H29	119.3
C5—C4—C3	121.5 (3)	C29—C30—C31	116.3 (3)
C5—C4—H4	119.3	C29—C30—N2	122.2 (3)
C3—C4—H4	119.3	C31—C30—N2	121.4 (3)
C4—C5—C6	116.5 (3)	C32—C31—C30	120.9 (3)
C4—C5—N1	121.3 (3)	C32—C31—H31	119.5
C6—C5—N1	122.2 (3)	C30—C31—H31	119.5
C7—C6—C5	121.1 (3)	C27—C32—C31	123.5 (3)
C7—C6—H6	119.5	C27—C32—H32	118.3
C5—C6—H6	119.5	C31—C32—H32	118.3
C6—C7—C2	122.9 (3)	N2—C33—H33A	109.5
C6—C7—H7	118.6	N2—C33—H33B	109.5
C2—C7—H7	118.6	H33A—C33—H33B	109.5
N1—C8—H8A	109.5	N2—C33—H33C	109.5
N1—C8—H8B	109.5	H33A—C33—H33C	109.5
H8A—C8—H8B	109.5	H33B—C33—H33C	109.5
N1—C8—H8C	109.5	N2—C34—H34A	109.5
H8A—C8—H8C	109.5	N2—C34—H34B	109.5
H8B—C8—H8C	109.5	H34A—C34—H34B	109.5
N1—C9—H9A	109.5	N2—C34—H34C	109.5
N1—C9—H9B	109.5	H34A—C34—H34C	109.5
H9A—C9—H9B	109.5	H34B—C34—H34C	109.5
N1—C9—H9C	109.5	C36—C35—C26	114.4 (3)
H9A—C9—H9C	109.5	C36—C35—H35A	108.7
H9B—C9—H9C	109.5	C26—C35—H35A	108.7
C11—C10—C1	113.8 (2)	C36—C35—H35B	108.7
C11—C10—H10A	108.8	C26—C35—H35B	108.7
C1—C10—H10A	108.8	H35A—C35—H35B	107.6
C11—C10—H10B	108.8	O3—C36—C37	120.1 (3)
C1—C10—H10B	108.8	O3—C36—C35	120.3 (3)
H10A—C10—H10B	107.7	C37—C36—C35	119.6 (3)
O1—C11—C12	119.9 (3)	C42—C37—C38	117.8 (3)
O1—C11—C12	119.9 (3)	C42—C37—C36	122.9 (3)
O1—C11—C10	120.0 (3)	C38—C37—C36	119.3 (3)
O1—C11—C10	120.0 (3)	C39—C38—C37	121.1 (4)
C12—C11—C10	120.2 (3)	C39—C38—H38	119.4
C17—C12—C13	119.0 (3)	C37—C38—H38	119.4
C17—C12—C11	118.6 (3)	C40—C39—C38	120.2 (4)
C13—C12—C11	122.3 (3)	C40—C39—H39	119.9
C14—C13—C12	120.6 (3)	C38—C39—H39	119.9
C14—C13—H13	119.7	C39—C40—C41	119.9 (4)
C12—C13—H13	119.7	C39—C40—H40	120.0
C13—C14—C15	119.3 (3)	C41—C40—H40	120.0
C13—C14—H14	120.3	C40—C41—C42	119.5 (4)
C15—C14—H14	120.3	C40—C41—H41	120.3
C16—C15—C14	120.6 (4)	C42—C41—H41	120.3
C16—C15—H15	119.7	C37—C42—C41	121.4 (3)
C14—C15—H15	119.7	C37—C42—H42	119.3
C15—C16—C17	120.0 (4)	C41—C42—H42	119.3
C15—C16—H16	120.0	C44—C43—C26	114.6 (2)
C17—C16—H16	120.0	C44—C43—H43A	108.6
C16—C17—C12	120.4 (3)	C26—C43—H43A	108.6
C16—C17—H17	119.8	C44—C43—H43B	108.6
C12—C17—H17	119.8	C26—C43—H43B	108.6
C19—C18—C1	116.0 (2)	H43A—C43—H43B	107.6
C19—C18—H18A	108.3	O4—C44—C45	120.3 (3)
C1—C18—H18A	108.3	O4—C44—C43	121.5 (3)
C19—C18—H18B	108.3	C45—C44—C43	118.2 (3)
C1—C18—H18B	108.3	C46—C45—C50	118.9 (3)
H18A—C18—H18B	107.4	C46—C45—C44	122.4 (3)
O2—C19—C20	120.8 (3)	C50—C45—C44	118.6 (3)
O2—C19—C18	121.3 (3)	C45—C46—C47	120.5 (3)
C20—C19—C18	117.9 (3)	C45—C46—H46	119.8
C21—C20—C25	118.2 (3)	C47—C46—H46	119.8
C21—C20—C19	118.8 (3)	C48—C47—C46	120.0 (4)
C25—C20—C19	123.1 (3)	C48—C47—H47	120.0
C22—C21—C20	121.6 (3)	C46—C47—H47	120.0
C22—C21—H21	119.2	C49—C48—C47	120.3 (4)
C20—C21—H21	119.2	C49—C48—H48	119.8
C23—C22—C21	119.9 (3)	C47—C48—H48	119.8
C23—C22—H22	120.1	CG1—C48—H48	179.5
C21—C22—H22	120.1	C48—C49—C50	120.6 (4)
C22—C23—C24	119.9 (3)	C48—C49—H49	119.7
C22—C23—H23	120.1	C50—C49—H49	119.7
C24—C23—H23	120.1	C49—C50—C45	119.7 (4)
C23—C24—C25	120.0 (3)	C49—C50—H50	120.2
C23—C24—H24	120.0	C45—C50—H50	120.2
C25—C24—H24	120.0		

Symmetry codes:  (i) −*x*+1, −*y*+1, −*z*.

### Hydrogen-bond geometry (Å, °)

**Table:**

*D*—H···*A*	*D*—H	H···*A*	*D*···*A*	*D*—H···*A*
C46—H46···O1	0.93	2.51	3.436 (4)	172
C23—H23···Cg2^ii^	0.93	2.67	3.535 (4)	155

Symmetry codes:  (ii) *x*, *y*+1, *z*.
